# Tacrolimus action pathways in an ointment base for hypertrophic scar prevention in a rabbit ear model^☆^^☆☆^

**DOI:** 10.1016/j.abd.2020.08.019

**Published:** 2021-05-15

**Authors:** Mariana Campos Souza Menezes, Marcelo Buzelin, Cristiana Buzelin Nunes, Luiz Ronaldo Alberti

**Affiliations:** a Instituto de Ensino e Pesquisa, Santa Casa de Belo Horizonte, Belo Horizonte, MG, Brazil; b Department of Pathological Anatomy, Faculdade de Medicina, Universidade Federal de Minas Gerais, Belo Horizonte, MG, Brazil; c Instituto de Ciências Biológicas e Saúde, Curso de Medicina, Centro Universitário de Belo Horizonte, Belo Horizonte, MG, Brazil; d Service of Pathology, Instituto Moacyr Junqueira, Belo Horizonte, MG, Brazil; e Department of Surgery, Universidade Federal de Minas Gerais, Belo Horizonte, MG, Brazil

**Keywords:** Hypertrophic scar, Keloid, Tacrolimus, Vascular endothelial growth factor, endocrine-gland-derived

## Abstract

**Background:**

Tacrolimus is used to prevent unaesthetic scars due to its action on fibroblast activity and collagen production modulation.

**Objectives:**

To evaluate the action pathways, from the histopathological point of view and in cytokine control, of tacrolimus ointment in the prevention of hypertrophic scars.

**Methods:**

Twenty-two rabbits were submitted to the excision of two 1-cm fragments in each ear, including the perichondrium. The right ear received 0.1% and 0.03% tacrolimus in ointment base twice a day in the upper wound and in the lower wound respectively. The left ear, used as the control, was treated with petrolatum. After 30 days, collagen fibers were evaluated using special staining, and immunohistochemistry analyses for smooth muscle actin, TGF-β and VEGF were performed.

**Results:**

The wounds treated with 0.1% tacrolimus showed weak labeling and a lower percentage of labeling for smooth muscle actin, a higher proportion of mucin absence, weak staining, fine and organized fibers for Gomori's Trichrome, strong staining and organized fibers for Verhoeff when compared to controls. The wounds treated with 0.03% tacrolimus showed weak labeling for smooth muscle actin, a higher proportion of mucin absence, strong staining for Verhoeff when compared to the controls. There was absence of TGF-β and low VEGF expression.

**Study limitations:**

The analysis was performed by a single pathologist. Second-harmonic imaging microscopy was performed in 2 sample areas of the scar.

**Conclusions:**

Both drug concentrations were effective in suppressing TGF-β and smooth muscle actin, reducing mucin, improving the quality of collagen fibers, and the density of elastic fibers, but only the higher concentration influenced elastic fiber organization.

## Introduction

The mechanism of healing, regardless of the causative agent, is systemic and dynamic and depends on the affected individual’s condition. It is characterized by a process of tissue reorganization and reconstitution, resulting from the interaction of biochemical, molecular and cellular events.^1^ When a causal agent, an injury, causes tissue damage and puts components of the extracellular matrix in contact with blood elements, it triggers the process called healing, culminating in the attraction of inflammatory cells into the affected site.^2^

The presence of mucin in the amorphous substance of a scar is usually associated with no good scar quality. Keloids can show a significant amount of mucin, whereas very often this substance is not observed in hypertrophic scars.^3^

Tacrolimus is an immunosuppressant drug, initially synthesized for systemic use aimed at preventing rejection in solid organ transplantation, which acts by binding to calmodulin and inhibiting calcineurin synthesis in lymphocytes, basophils, dendritic cells, and eosinophils.^4^, ^5^ Therefore, it inhibits the transcription of some inflammatory cytokines, such as IL-2, IL-3, IL-4, TNF-alpha, and the activation of T lymphocytes, reduces the population of dendritic cells, which are antigen-presenting and can trigger an allergic inflammatory response, reduces mast cell and basophil mediators (histamine), reduces pruritus by decreasing the production of substance P and neuronal growth factor.^5^, ^6^ It shows less skin absorption compared to glucocorticoids, which increases its safety of use, due to the lower potential for systemic adverse effects.^7^, ^8^

This is a study about its topical use, on unaesthetic scars, to investigate whether it would promote apoptosis and suppression of fibroblast activity.^6^

## Methods

The study was carried out according to the UK Animals (Scientific Procedures) Act, 1986, National Institutes of Health Guide for the Care and Use of Laboratory Animals (1978), and the Brazilian Animal Experimentation Code (1988) and was approved by the Ethical Committee for Animal Use of Universidade Federal de Minas Gerais, protocol n. 74/2016.^9^, ^10^, ^11^

**Figure 1:**
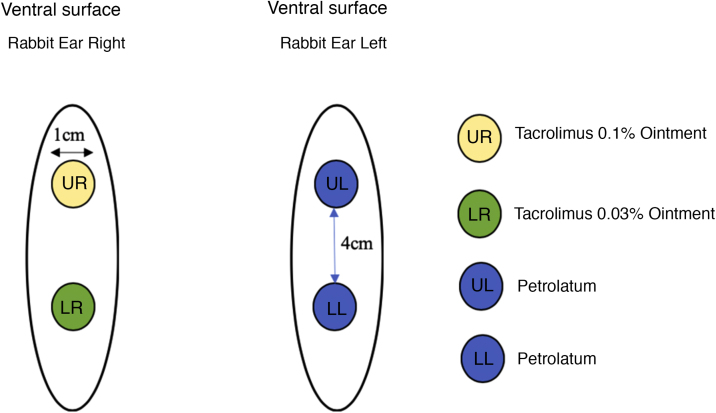
Wounds treated with petrolatum and tacrolimus applied in the ointment at 0.1% and 0.03%.

Twenty-two male New Zealand white rabbits (*Oryctolagus cuniculus*), aged approximately two months old, identified by numbers, were used in the study. The animals were submitted to the excision of two 1-cm fragments on the ventral surface of each ear, 4 cm apart, up to the cartilage, including the perichondrium. The right ear was treated with tacrolimus; 0.1% in ointment base applied to the upper wound and 0.03% in ointment base to the lower wound, both twice a day. The left ear, used as the control, was treated with petrolatum at the same dose as the the medication under study (Fig. 1).

On the 30^th^ postoperative day, the wounds were excised and prepared for histopathological analysis on slides stained with Hematoxylin & Eosin, Alcian blue, Gomori’s trichrome, and Verhoeff stain and for immunohistochemical analysis with smooth muscle actin, TGF-β, and VEGF antibodies. Some variables were evaluated (Table 1, Table 2).

**Table 1:** Microscopic wound assessment, special staining methods, and used criteria.

	Microscopic evaluation of wounds		
	Alcian Blue	Verhoeff stain	Gomori’s trichrome
**Staining intensity**	0 – Absent	1 – Weak	1 – Weak
	1 – Weak	2 – Medium	2 – Strong
	2 – Strong	3 – Strong	
**Fiber orientation**	–	1 – Organized	1 – Organized
		2 – Disorganized	2 – Disorganized
**Fiber thickness**	–	–	1 – Thin
			2 – Thick

**Table 2:** Immunohistochemical wound assessment and used criteria.

	Immunohistochemical wound assessment		
	Muscle actin	VEGF	TGF β
**Percentage of stained wound**	0%–100%	0%–100%	0%–100%
**Staining intensity**	1 – Weak	0 – Absent	0 – Absent
	2 – Medium	1 – Weak	1 – Weak
	3 – Strong	2 – Medium	2 – Strong
		3 – Strong	3 – Strong

The slides stained for Hematoxylin & Eosin were sent for image analysis by second-harmonic imaging microscopy and two-photon excitation fluorescence microscopy for collagen fiber analysis, in two randomly determined square areas measuring 500 µm.

The PyFibre program was used to perform image analysis and calculations to generate some metrics about collagen fibers:

Global anisotropy (0–1): When the fibers are randomly distributed, the anisotropy value is equal to 0 and when the fibers are oriented in the same direction, the anisotropy value is equal to 1.

Fiber wavy measurement (0–1): The more wavy the fiber is, the closer to zero is the value.

Linearity of the fiber segments (0–1): value = 0 (zero) for a completely circular fiber and value = 1 for a completely linear one.

Density of fibers and cells: image intensity in the imaged area, for both fibers and cells.

A significance level of 5% was used in all tests; therefore, differences are significant considering p ≤ 0.05. The software used for the analyses was SPSS for Windows, version 20 (SPSS Inc., Chicago, IL, United States).

## Results

None of the animals involved in the experiment showed skin alterations under ectoscopy throughout the experiment. They showed good general status and the recovery from the surgical intervention was favorable and spontaneous, in a controlled environment. Two rabbits died during the anesthetic induction. The other animals survived the thirty days of the experiment.

**Figure 2:**
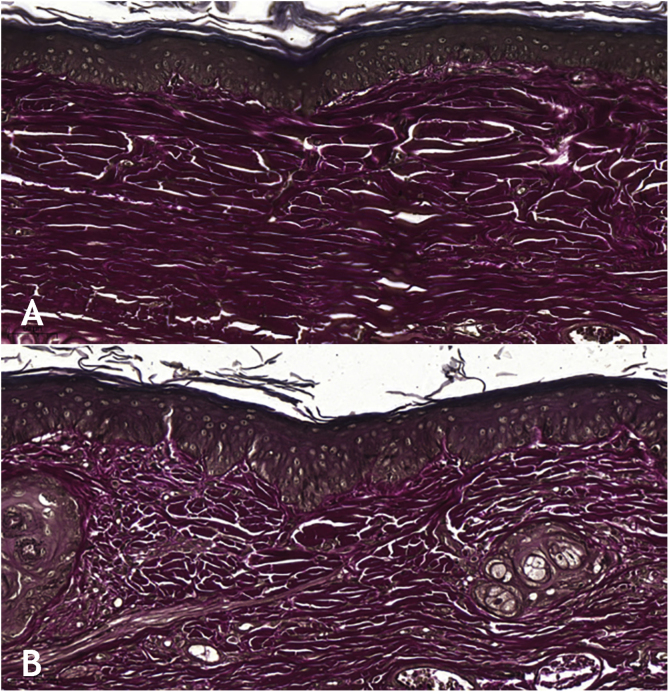
(A), Wound treated with 0.1% tacrolimus, showing strong staining and organized elastic fibers (Verhoeff, ×20). (B), Control wound showing weak staining and disorganized elastic fibers (Verhoeff, ×20).

**Figure 3:**
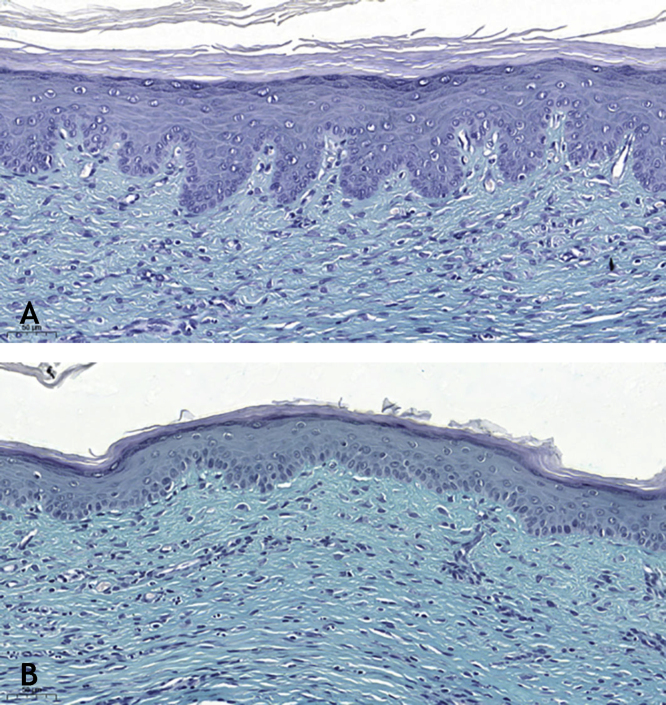
(A), Wound treated with 0.03 % tacrolimus, absence of mucin. (Alcian blue, ×20). (B), Control wound showing a higher amount of mucin. (Alcian blue, ×20).

Microscopic analyses were performed without loss of wound fragments and the slides were analyzed under an optical microscope at 20× and 40× magnifications. The classifications were performed according to the proposed standardization. Considering the variables for collagen: Anisotropy and Fiber Density, the group that used the higher dose of the medication, showed a significantly lower mean than the group that used the lower dose (p = 0.036 and p = 0.027). Wounds treated with the drug at the higher concentration showed a higher proportion of weak labeling for smooth muscle actin and a lower percentage of labeling when compared to their control (p = 0.005 and p = 0.034). Wounds in which tacrolimus was used (0.1%) showed a higher proportion of mucin absence (p = 0.029), weak staining intensity, fine and organized fibers with Gomori’s trichrome (p = 0.047, p = 0.025 and p = 0.004), strong staining intensity and organized fibers with Verhoeff’s stain (Fig. 2), (p < 0.001 and p < 0.001) when compared to controls. A higher proportion of mucin absence (Fig. 3), (p = 0.002) stronger staining intensity for Verhoeff’s stain, (p < 0.001) was observed in the wounds treated with the drug at 0.03% concentration compared to controls.

**Figure 4:**
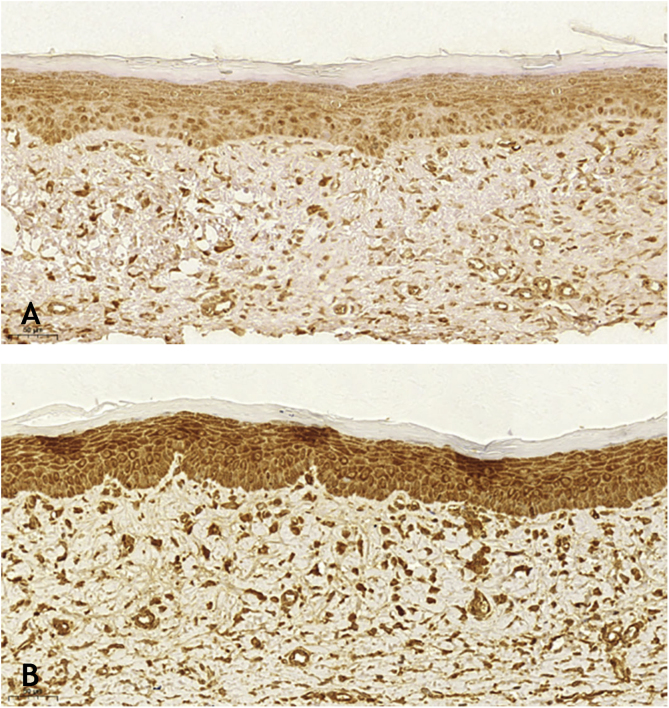
Immunohistochemistry analysis for smooth muscle actin: (A), Wound treated with 0.03% tacrolimus, showing weak labeling (×20). (B), Control wound showing strong labeling (×20).

Wounds treated with 0.03% tacrolimus had a higher proportion of weak labeling for smooth muscle actin when compared to their control (Fig. 4), (p = 0.026). Wounds treated with 0.1% tacrolimus had a higher proportion of strong staining intensity and organized fibers for Verhoeff’s stain when compared to wounds treated with 0.03% medication (p = 0.040 and p = 0.027).

## Discussion

Tacrolimus in ointment base is not a formally indicated drug for the treatment of keloids and hypertrophic scars, due to the insufficient number of studies carried out for this purpose.^12^

Unaesthetic scars usually appear one month after the causing injury and histopathological alterations, such as neovascularization, increased collagen and fibroblast deposition, occur in the dermis, generating a chronic inflammatory process, especially in the reticular dermis.^13^ Epidermal injuries hardly ever result in unaesthetic scars, and such scars are more commonly seen in more exposed areas and those under more tension.^14^

The sampling was calculated based on previously published studies, using the same experimental model, and taking into account a 25% loss, which is predicted for such a model. The fact that measures based on protection norms and laws regarding animal experimentation were adopted, justifies the fact that the loss observed in the present study is less than ten percent.^15^

In 2007, Mustoe suggested the model for reproducing hypertrophic scars on the back of rabbit ears and a follow-up and observation period of approximately thirty days, a period adopted in other experiments, based on the premise that unaesthetic scars usually manifest one month after the injury.^16^, ^17^, ^18^, ^19^ This model has been used ever since, including for the study of tacrolimus, but using the intradermal injectable formulation, with positive results in unaesthetic scars. This knowledge and the easy access and cost of the medication in ointment form at 0.1% and 0.03% concentrations, furthered the present study.^15^

The use of tacrolimus culminated in scars with a higher proportion of finer and organized collagen fibers under Gomori's trichrome stain. Wounds treated with tacrolimus at the higher concentration showed a lower mean density of collagen fibers (p = 0.027), and the anisotropy of these fibers (p = 0.036), when compared to those treated with tacrolimus (0.03%), which indicates that the collagen fibers, in addition to being less dense, are distributed in a less parallel manner in wounds treated with the 0.1% formulation. The use of 0.1% tacrolimus resulted in a higher proportion of weak staining with Gomori’s trichrome, with finer and more organized fibers when compared to the control, which is in agreement with the findings of second-harmonic imaging microscopy and two-photon excitation fluorescence microscopy. These results are in accordance with the literature since in unaesthetic scars, the collagen fibers are arranged in a more parallel manner when compared to normal skin, the collagen bundles are thicker, more widely spaced and the fibroblasts produce excess collagen, mainly type III collagen.^20^ Keloids have a large amount of disorganized collagen and mucopolysaccharides in the extracellular matrix. In the hypertrophic scar, collagen is more often deposited in the superficial layers of the reticular dermis, whereas the collagen extends throughout the reticular dermis in keloids.^21^, ^22^

When analyzing the immunohistochemistry results, the wounds on the right ear, regardless of tacrolimus concentration, showed a higher proportion of weak labeling for smooth muscle actin, abscence of labeling for TGF-β (p < 0.001 and p = 0.030, respectively), and a lower percentage of labeling for smooth muscle actin (p = 0.008). Wounds treated with 0.1% and 0.03% tacrolimus also showed a higher proportion of weak labeling for smooth muscle actin, when compared to the respective controls (p = 0.005 and p = 0.026) and those treated with the highest drug concentration showed a lower percentage of labeling for smooth muscle actin when compared to its control (p = 0.034). That is, the use of the assessed drug, at the two concentrations, probably caused suppression of the expression of TGF-β and, thus, of smooth muscle actin. These findings are justified by the fact that hypertrophic and keloid scars show an imbalance in the release of local growth factors such as TGF-β, which stimulates the production of extracellular matrix and has fibrogenic action.^23^, ^24^, ^25^

TGF-β 1 and -2 stimulate the appearance of myofibroblasts rich in smooth muscle alpha-actin and exert a powerful stimulus for alpha-actin synthesis.^26^ Myofibroblasts play an important role in organogenesis, repair, inflammation and fibrosis through the secretion of inflammatory cytokines, chemokines, growth factors, and inflammatory mediators. When present in cicatricial wounds, myofibroblasts express the smooth muscle phenotype only temporarily; in unaesthetic scars, this phenotype persists, as well as in diseases with contraction of fibers and neoplastic proliferative conditions.^27^

Fibroblasts present in unaesthetic scars show increased sensitivity and impaired negative feedback in relation to TGF-β, resulting in greater collagen deposition.^28^, ^29^ The use of the drug at its lower concentration resulted in wounds with a greater proportion of strong labeling for VEGF, when compared to the use of the drug at a higher concentration; however, there was no difference between the control and treatment groups.

The cytokine in question is produced by fibroblasts and its synthesis is increased in keloids.^30^ A study that used the same rabbit ear model, showed that acting on angiogenesis in a preventive manner to prevent excess is effective to avoid unaesthetic scars.^31^ Endothelial dysfunction, with a consequent increase in vascular permeability and accumulation of inflammatory cells and inflammatory factors in the injury area, promoting an increase in fibroblast activity, favors the appearance of unaesthetic scars.

Angiogenesis is stimulated when there is tension in the scar; systemic factors that promote vasodilation predispose to unfavorable scarring and it is known that several therapies for pathological scars are based on the suppression of blood vessels.^32^ VEGF levels are higher in deeper injuries when compared to more superficial ones and normal skin.^33^ Based on the abovementioned facts, tacrolimus at a higher concentration resulted in wounds with a lower VEGF expression, although a previous study with this medication showed a higher concentration of capillaries in wounds treated with this concentration of the drug.^15^

Evidence shows that capillaries formed in the angiogenesis phase are essential for tissue regeneration, but an exacerbation in this phase is associated with fibrotic scars.^34^, ^35^ Most likely, the higher number of capillaries in wounds treated with 0.1% tacrolimus did not represent an exacerbated angiogenesis, but only variations within the normal range.

Mucin is rarely seen in hypertrophic scars; however, it is often abundant in the extracellular matrix of keloid scars.^6^ Tracolimus, regardless of the concentration used, resulted in scars with a higher proportion of absence of mucin, when compared to the control (p = 0.001), as the two concentrations, when analyzed alone, also showed a higher proportion of absence of mucin, when compared to the respective controls (p = 0.029 and p = 0.002). These findings are in accordance with the literature, corroborating the hypothesis that the drug acts on the extracellular matrix constitution, preventing of unaesthetic scars.

The use of tacrolimus, regardless of its concentration, has resulted in a higher proportion of strong staining with Verhoeff and a higher proportion of more organized elastic fibers in the scars (p < 0.001 and p < 0.001). The same can be observed when analyzing the use of tacrolimus at the higher concentration compared to its control (p < 0.001). The use of the drug at a lower concentration, on the other hand, resulted in wounds with a higher proportion of strong staining with Verhoeff, without changes in fiber organization, when compared to the control (p < 0.001); that is, the use of the drug increased elastic fiber density, but only the higher concentration of the drug acted on their organization.

The findings described herein can be justified by the fact that elastic fibers, when present, dense and well distributed, give quality to the scar and, in many cases, are absent in keloids and present, although at lesser amounts, in hypertrophic scars.^3^ Therefore, the two concentrations of the studied drug have a favorable effect on elastic fibers during healing, although when comparing wounds treated with 0.1% tacrolimus with those treated with 0.03% tacrolimus, there was a higher proportion of strong staining and organized fibers after Verhoeff staining in the first ones (p = 0.040 and p = 0.027). That is, the drug at a higher concentration contributes more to the quality of elastic fibers in the scars.

A higher proportion of the healing process was present in the most superficial layers of the skin in the wounds treated with tacrolimus at 0.03% when compared to its control (p = 0.007); however, this difference occurred due to the control group. Unaesthetic scars have most of their histopathological alterations, extracellular matrix composition, and inflammatory process located in the deeper layers of the dermis.^13^, ^36^, ^37^, ^38^, ^39^ The incisions made in the present experiment all had the same dimensions and reached the same depth, thus having the same healing potential.

## Conclusions

Tacrolimus acted on the TGF-β and smooth muscle actin pathway, reducing their expressions in the treated wounds. It improved the quality of the scars by improving the extracellular matrix composition, with a reduction in mucin and gain in the collagen fiber quality, which were thinner and more organized, whereas the elastic fibers were more dense and organized. The 0.1% concentration was more effective in suppressing smooth muscle actin. It resulted in wounds with less dense, more organized, and finer collagen fibers, as well as more organized elastic fibers.

The 0.03% concentration resulted in scars with a more superficially distributed inflammatory process. The use of tacrolimus in ointment base to prevent hypertrophic scars is a promising option in an experimental model. Since no systemic or local adverse effects were observed with either of the two drug concentrations used, and considering the proven benefits, the next step would be a study in humans.

## Financial support

None declared.

## Authors’ contributions

Mariana Campos Souza Menezes: Performing the experiment; development of the research structure; data analysis; writing of the manuscript.

Marcelo Buzelin: Immunohistochemistry analysis and development of the research structure.

Cristiana Buzelin Nunes: Development of the research structure; review of the manuscript writing and data analysis.

Luiz Ronaldo Alberti: Development of the research structure; review of the manuscript writing and data analysis.

## Conflicts of interest

None declared.
